# *catena*-Poly[[diaqua­(1,10-phenanthroline-κ^2^ *N*,*N*′)nickel(II)]-μ-1*H*-benzimidazole-5,6-dicarboxyl­ato-κ^2^ *N* ^3^:*O* ^6^]

**DOI:** 10.1107/S1600536809019680

**Published:** 2009-05-29

**Authors:** Wen-Dong Song, Hao Wang, Shi-Wei Hu, Pei-Wen Qin, Shi-Jie Li

**Affiliations:** a College of Science, Guang Dong Ocean University, Zhanjiang 524088, People’s Republic of China

## Abstract

In the title complex, [Ni(C_9_H_4_N_2_O_4_)(C_12_H_8_N_2_)(H_2_O)_2_]_*n*_, the Ni^II^ atom is hexa­coordinated by one N and one O atom from two different 1*H*-benzimidazole-5,6-dicarboxyl­ate ligands, two N atoms from one 1,10-phenanthroline ligand and two water mol­ecules. The flexible 1*H*-benzimidazole-5,6-dicarboxyl­ate ligands link the Ni^II^ centres, forming an infinite zigzag chain parallel to [001]. The crystal packing is governed by inter­molecular hydrogen-bonding inter­actions of the O—H⋯O, N—H⋯O and C—H⋯O types.

## Related literature

For background to 1*H*-benzoimidazole-5,6-dicarboxyl­ate complexes, see: Lo *et al.* (2007 ▶); Yao *et al.* (2008 ▶); Gao *et al.* (2008 ▶). For background to 1,10-phenanthroline complexes, see: Chesnut *et al.* (1999 ▶).
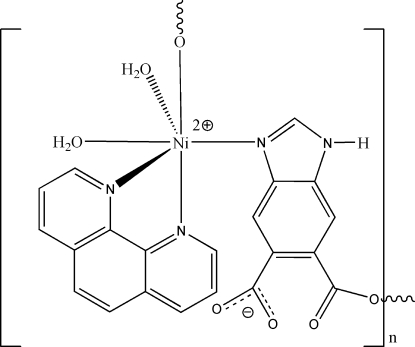


## Experimental

### 

#### Crystal data

[Ni(C_9_H_4_N_2_O_4_)(C_12_H_8_N_2_)(H_2_O)_2_]
*M* *_r_* = 479.09
Monoclinic, 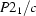

*a* = 10.021 (2) Å
*b* = 16.980 (3) Å
*c* = 15.327 (5) Å
β = 129.09 (2)°
*V* = 2024.3 (9) Å^3^
*Z* = 4
Mo *K*α radiation
μ = 1.01 mm^−1^
*T* = 293 K
0.31 × 0.26 × 0.22 mm

#### Data collection

Rigaku/MSC Mercury CCD diffractometer
Absorption correction: multi-scan (*REQAB*; Jacobson, 1998 ▶) *T*_min_ = 0.746, *T*_max_ = 0.809
15765 measured reflections
3639 independent reflections
3195 reflections with *I* > 2σ(*I*)
*R*_int_ = 0.039

#### Refinement

*R*[*F* ^2^ > 2σ(*F* ^2^)] = 0.032
*wR*(*F* ^2^) = 0.091
*S* = 1.09
3639 reflections
289 parameters
H-atom parameters constrained
Δρ_max_ = 0.37 e Å^−3^
Δρ_min_ = −0.25 e Å^−3^

### 

Data collection: *RAPID-AUTO* (Rigaku, 1998 ▶); cell refinement: *RAPID-AUTO*; data reduction: *CrystalStructure* (Rigaku/MSC, 2002 ▶); program(s) used to solve structure: *SHELXS97* (Sheldrick, 2008 ▶); program(s) used to refine structure: *SHELXL97* (Sheldrick, 2008 ▶); molecular graphics: *ORTEPII* (Johnson, 1976 ▶); software used to prepare material for publication: *SHELXL97*.

## Supplementary Material

Crystal structure: contains datablocks I, global. DOI: 10.1107/S1600536809019680/im2119sup1.cif

Structure factors: contains datablocks I. DOI: 10.1107/S1600536809019680/im2119Isup2.hkl

Additional supplementary materials:  crystallographic information; 3D view; checkCIF report

## Figures and Tables

**Table 1:** Hydrogen-bond geometry (Å, °)

*D*—H⋯*A*	*D*—H	H⋯*A*	*D*⋯*A*	*D*—H⋯*A*
O1*W*—H1*W*⋯O1^i^	0.84	1.90	2.710 (2)	163
O1*W*—H2*W*⋯O4^ii^	0.84	1.76	2.584 (2)	165
O2*W*—H3*W*⋯O1^i^	0.84	1.87	2.703 (2)	169
O2*W*—H4*W*⋯O1^ii^	0.84	2.11	2.932 (2)	165
N2—H2⋯O2^iii^	0.86	2.00	2.739 (2)	144
N2—H2⋯O1^iii^	0.86	2.54	3.355 (2)	159
C10—H10⋯O2^ii^	0.93	2.56	3.346 (8)	143

Symmetry codes: (i) 
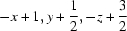
; (ii) 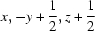; (iii) 
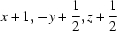
.

**Fig. 1.**
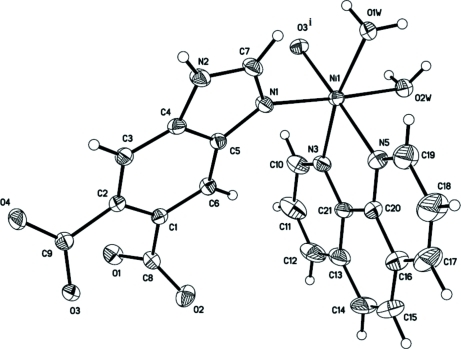
The structure of the title compound, showing the atomic numbering scheme. Non-H atoms are shown with 30% probability displacement ellipsoids. [Symmetry codes: (i) x, 1/2 - y, 1/2 + z.

**Fig. 2.**
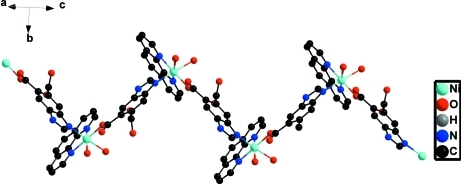
A view of the infinite chain of title compound.

**Fig. 3.**
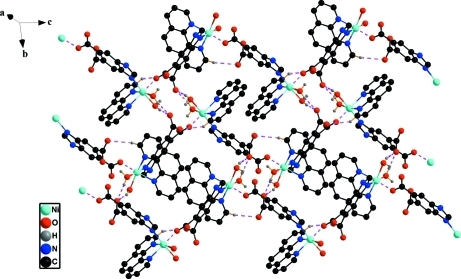
A view of the two-dimensional layer constructed by O—H···O and C—H···O hydrogen bonding interactions.

## supplementary crystallographic information

### Comment

In the structural investigation of 1*H*-benzimidazole-5,6-dicarboxylate complexes, it has been found that 1*H*-benzimidazole-5,6-dicarboxylic acid can function as a multidentate ligand (Lo *et al.*, 2007; Yao *et al.*, 2008; Gao *et al.*, 2008), with versatile binding and coordination modes. 1,10-Phenanthroline is also a good example for a bridging ligand that can link metal centres into extended networks, and a number of one-, two- and three- dimensional metal-1,10-phenanthroline frameworks have been generated (Chesnut *et al.*, 1999). The reaction of 1*H*-benzimidazole-5,6-dicarboxylic acid with nickel chloride in an alkaline aqueous solution yielded a new Ni^II^ coordination polymer, whose crystal structure is reported here.

As illustrated in Figure 1, the Ni^II^ atom exhibits a slightly distorted octahedral coordination sphere, defined by one N and one O atom from two different 1*H*-benzimidazole-5,6-dicarboxylate ligands, two N atoms from one 1,10-phenanthroline ligand and two water molecules. The metal atoms are linked by bidentate 1*H*-benzimidazole-5,6-dicarboxylate groups into a linear chain (Fig. 2). Inter/intramolecular O—H···O and C—H···O hydrogen bonds between the carboxylate O atoms of 1*H*-benzimidazole-5,6-dicarboxylate and the coordinated water molecule lead to a two-dimensional layer (Fig. 3). The layers are further self-assembled into a three-dimensional supramolecular network by intermolecular N—H···O hydrogen bonds between the imidazole units and carboxylate groups (Table 1).

### Experimental

A mixture of nickel chloride (1 mmol), 1*H*-benzimidazole-5,6-dicarboxylic acid (1 mmol), 1,10-phenanthroline (1 mmol), NaOH (1.5 mmol) and H_2_O (12 ml) was placed in a 23 ml Teflon reactor, which was heated to 433 K for three days and then cooled to room temperature at a rate of 10 K h^-1^. The crystals obtained were washed with water and dryed in air.

### Refinement

Carbon and nitrogen bound H atoms were placed at calculated positions and were treated as riding on the parent C or N atoms with C—H = 0.93 Å, N—H = 0.86 Å, and with *U*_iso_(H) = 1.2 *U*_eq_(C, N). The water H-atoms were located in a difference map, and were refined with a distance restraint of O—H = 0.84 Å; their *U*_iso_ values were refined.

### Crystal data

**Table:**

[Ni(C_9_H_4_N_2_O_4_)(C_12_H_8_N_2_)(H_2_O)_2_]	*F*(000) = 984
*M**_r_* = 479.09	*D*_x_ = 1.572 Mg m^−^^3^
Monoclinic, *P*2_1_/*c*	Mo *K*α radiation, λ = 0.71073 Å
Hall symbol: -P 2ybc	Cell parameters from 3600 reflections
*a* = 10.021 (2) Å	θ = 1.4–28°
*b* = 16.980 (3) Å	µ = 1.01 mm^−^^1^
*c* = 15.327 (5) Å	*T* = 293 K
β = 129.09 (2)°	Block, blue
*V* = 2024.3 (9) Å^3^	0.31 × 0.26 × 0.22 mm
*Z* = 4	

### Data collection

**Table:**

Rigaku/MSC Mercury CCD diffractometer	3639 independent reflections
Radiation source: fine-focus sealed tube	3195 reflections with *I* > 2σ(*I*)
graphite	*R*_int_ = 0.039
ω scans	θ_max_ = 25.2°, θ_min_ = 3.2°
Absorption correction: multi-scan (REQAB; Jacobson, 1998)	*h* = −11→12
*T*_min_ = 0.746, *T*_max_ = 0.809	*k* = −20→20
15765 measured reflections	*l* = −18→18

### Refinement

**Table:**

Refinement on *F*^2^	Primary atom site location: structure-invariant direct methods
Least-squares matrix: full	Secondary atom site location: difference Fourier map
*R*[*F*^2^ > 2σ(*F*^2^)] = 0.032	Hydrogen site location: inferred from neighbouring sites
*wR*(*F*^2^) = 0.091	H-atom parameters constrained
*S* = 1.09	*w* = 1/[σ^2^(*F*_o_^2^) + (0.0565*P*)^2^ + 0.2141*P*] where *P* = (*F*_o_^2^ + 2*F*_c_^2^)/3
3639 reflections	(Δ/σ)_max_ = 0.001
289 parameters	Δρ_max_ = 0.37 e Å^−^^3^
0 restraints	Δρ_min_ = −0.25 e Å^−^^3^

### Special details

**Table:**

Geometry. All e.s.d.'s (except the e.s.d. in the dihedral angle between two l.s. planes) are estimated using the full covariance matrix. The cell e.s.d.'s are taken into account individually in the estimation of e.s.d.'s in distances, angles and torsion angles; correlations between e.s.d.'s in cell parameters are only used when they are defined by crystal symmetry. An approximate (isotropic) treatment of cell e.s.d.'s is used for estimating e.s.d.'s involving l.s. planes.
Refinement. Refinement of *F*^2^ against ALL reflections. The weighted *R*-factor *wR* and goodness of fit *S* are based on *F*^2^, conventional *R*-factors *R* are based on *F*, with *F* set to zero for negative *F*^2^. The threshold expression of *F*^2^ > σ(*F*^2^) is used only for calculating *R*-factors(gt) *etc*. and is not relevant to the choice of reflections for refinement. *R*-factors based on *F*^2^ are statistically about twice as large as those based on *F*, and *R*- factors based on ALL data will be even larger.

### Fractional atomic coordinates and isotropic or equivalent isotropic displacement parameters (Å^2^)

**Table:**

	*x*	*y*	*z*	*U*_iso_*/*U*_eq_	
Ni1	0.55530 (3)	0.434786 (13)	0.82246 (2)	0.02591 (11)	
O1	0.38387 (19)	0.10540 (8)	0.49825 (13)	0.0398 (4)	
O1W	0.76448 (17)	0.50597 (8)	0.94690 (12)	0.0318 (3)	
H1W	0.7283	0.5441	0.9617	0.048*	
H2W	0.8137	0.4736	1.0003	0.048*	
O2	0.27333 (18)	0.22040 (9)	0.41361 (14)	0.0456 (4)	
O2W	0.39504 (19)	0.49621 (9)	0.84828 (13)	0.0395 (4)	
H3W	0.4521	0.5341	0.8920	0.059*	
H4W	0.3796	0.4618	0.8805	0.059*	
O3	0.59904 (17)	0.14247 (8)	0.43858 (11)	0.0314 (3)	
O4	0.86749 (19)	0.10944 (10)	0.58603 (13)	0.0479 (4)	
N1	0.7201 (2)	0.37301 (10)	0.80627 (14)	0.0296 (4)	
N2	0.9708 (2)	0.34131 (11)	0.84962 (15)	0.0357 (4)	
H2	1.0793	0.3421	0.8837	0.043*	
N3	0.3234 (2)	0.38391 (10)	0.68890 (14)	0.0334 (4)	
N5	0.4878 (3)	0.51071 (11)	0.69453 (16)	0.0407 (4)	
C1	0.5577 (2)	0.22029 (11)	0.58394 (16)	0.0259 (4)	
C2	0.7187 (2)	0.20098 (11)	0.61273 (16)	0.0277 (4)	
C3	0.8679 (2)	0.23640 (12)	0.70388 (17)	0.0300 (4)	
H3	0.9750	0.2227	0.7257	0.036*	
C4	0.8508 (2)	0.29313 (12)	0.76130 (16)	0.0288 (4)	
C5	0.6930 (2)	0.31336 (11)	0.73392 (16)	0.0261 (4)	
C6	0.5439 (2)	0.27566 (11)	0.64394 (17)	0.0273 (4)	
H6	0.4378	0.2875	0.6248	0.033*	
C7	0.8876 (3)	0.38666 (13)	0.87258 (18)	0.0355 (5)	
H7	0.9419	0.4241	0.9296	0.043*	
C8	0.3932 (2)	0.17945 (12)	0.48969 (17)	0.0296 (4)	
C9	0.7307 (2)	0.14601 (11)	0.54114 (17)	0.0297 (4)	
C10	0.2431 (3)	0.32210 (15)	0.6884 (2)	0.0456 (6)	
H10	0.2925	0.2953	0.7554	0.055*	
C11	0.0856 (3)	0.29550 (19)	0.5899 (3)	0.0632 (8)	
H11	0.0322	0.2514	0.5915	0.076*	
C12	0.0117 (3)	0.33512 (19)	0.4919 (2)	0.0622 (8)	
H12	−0.0927	0.3178	0.4263	0.075*	
C13	0.0909 (3)	0.40118 (17)	0.4888 (2)	0.0523 (7)	
C14	0.0237 (4)	0.4466 (2)	0.3894 (2)	0.0712 (9)	
H14	−0.0821	0.4332	0.3217	0.085*	
C15	0.1116 (5)	0.5081 (2)	0.3928 (2)	0.0775 (10)	
H15	0.0667	0.5352	0.3268	0.093*	
C16	0.2709 (4)	0.53257 (17)	0.4943 (2)	0.0600 (7)	
C17	0.3695 (5)	0.59590 (19)	0.5046 (3)	0.0785 (10)	
H17	0.3296	0.6257	0.4415	0.094*	
C18	0.5209 (5)	0.61432 (19)	0.6043 (3)	0.0812 (10)	
H18	0.5865	0.6558	0.6099	0.097*	
C19	0.5781 (4)	0.57061 (14)	0.6991 (3)	0.0577 (7)	
H19	0.6827	0.5836	0.7678	0.069*	
C20	0.3383 (3)	0.49073 (13)	0.59409 (19)	0.0406 (5)	
C21	0.2485 (3)	0.42380 (14)	0.59078 (19)	0.0391 (5)	

### Atomic displacement parameters (Å^2^)

**Table:**

	*U*^11^	*U*^22^	*U*^33^	*U*^12^	*U*^13^	*U*^23^
Ni1	0.02516 (17)	0.02713 (17)	0.02261 (17)	0.00032 (8)	0.01370 (14)	0.00058 (9)
O1	0.0485 (9)	0.0309 (8)	0.0463 (9)	−0.0124 (6)	0.0330 (8)	−0.0050 (6)
O1W	0.0337 (8)	0.0273 (7)	0.0319 (8)	−0.0008 (5)	0.0196 (7)	−0.0031 (6)
O2	0.0250 (8)	0.0409 (9)	0.0475 (10)	0.0004 (6)	0.0117 (8)	−0.0029 (7)
O2W	0.0397 (8)	0.0405 (8)	0.0361 (9)	0.0052 (6)	0.0228 (8)	−0.0012 (6)
O3	0.0311 (7)	0.0347 (7)	0.0257 (7)	0.0014 (5)	0.0165 (7)	−0.0032 (6)
O4	0.0298 (8)	0.0577 (10)	0.0384 (9)	0.0097 (7)	0.0130 (7)	−0.0155 (8)
N1	0.0280 (9)	0.0324 (9)	0.0272 (9)	−0.0026 (6)	0.0168 (8)	−0.0065 (7)
N2	0.0222 (8)	0.0463 (10)	0.0297 (9)	−0.0029 (7)	0.0121 (8)	−0.0118 (8)
N3	0.0301 (9)	0.0417 (10)	0.0281 (9)	0.0000 (7)	0.0181 (8)	−0.0045 (7)
N5	0.0483 (11)	0.0373 (10)	0.0342 (10)	0.0045 (8)	0.0250 (10)	0.0062 (8)
C1	0.0247 (10)	0.0249 (9)	0.0250 (10)	−0.0008 (7)	0.0141 (9)	0.0011 (7)
C2	0.0266 (10)	0.0275 (10)	0.0249 (10)	0.0023 (7)	0.0142 (9)	0.0004 (8)
C3	0.0238 (9)	0.0356 (11)	0.0280 (10)	0.0020 (8)	0.0150 (9)	−0.0013 (8)
C4	0.0247 (9)	0.0324 (10)	0.0234 (10)	−0.0004 (7)	0.0123 (9)	−0.0004 (8)
C5	0.0273 (9)	0.0259 (9)	0.0253 (10)	0.0003 (7)	0.0167 (9)	0.0006 (8)
C6	0.0227 (9)	0.0301 (10)	0.0306 (11)	−0.0013 (7)	0.0175 (9)	−0.0022 (8)
C7	0.0290 (11)	0.0405 (12)	0.0309 (11)	−0.0045 (8)	0.0159 (10)	−0.0123 (9)
C8	0.0283 (10)	0.0314 (11)	0.0331 (11)	−0.0057 (8)	0.0212 (9)	−0.0058 (8)
C9	0.0251 (10)	0.0316 (10)	0.0296 (11)	−0.0026 (8)	0.0160 (9)	−0.0027 (8)
C10	0.0447 (13)	0.0568 (15)	0.0428 (13)	−0.0148 (11)	0.0311 (12)	−0.0131 (11)
C11	0.0558 (16)	0.084 (2)	0.0632 (19)	−0.0333 (15)	0.0437 (16)	−0.0301 (16)
C12	0.0387 (14)	0.091 (2)	0.0433 (16)	−0.0135 (13)	0.0195 (13)	−0.0268 (15)
C13	0.0389 (13)	0.0677 (17)	0.0340 (13)	0.0058 (12)	0.0152 (12)	−0.0118 (12)
C14	0.0542 (18)	0.091 (2)	0.0265 (14)	0.0131 (15)	0.0055 (14)	−0.0051 (13)
C15	0.085 (2)	0.079 (2)	0.0324 (15)	0.0178 (18)	0.0202 (16)	0.0141 (14)
C16	0.0776 (19)	0.0562 (16)	0.0387 (14)	0.0174 (14)	0.0330 (15)	0.0119 (12)
C17	0.110 (3)	0.0605 (19)	0.056 (2)	0.0062 (18)	0.048 (2)	0.0265 (15)
C18	0.112 (3)	0.0601 (19)	0.069 (2)	−0.0128 (18)	0.056 (2)	0.0185 (16)
C19	0.0712 (19)	0.0452 (15)	0.0536 (17)	−0.0087 (12)	0.0378 (16)	0.0068 (11)
C20	0.0473 (13)	0.0392 (12)	0.0311 (12)	0.0119 (9)	0.0227 (11)	0.0060 (9)
C21	0.0332 (12)	0.0501 (13)	0.0249 (11)	0.0109 (9)	0.0140 (10)	−0.0035 (9)

### Geometric parameters (Å, °)

**Table:**

Ni1—O3^i^	2.0241 (14)	C2—C9	1.502 (3)
Ni1—N5	2.0715 (19)	C3—C4	1.386 (3)
Ni1—N3	2.0828 (18)	C3—H3	0.9300
Ni1—N1	2.0994 (16)	C4—C5	1.399 (3)
Ni1—O1W	2.1098 (15)	C5—C6	1.395 (3)
Ni1—O2W	2.1507 (15)	C6—H6	0.9300
O1—C8	1.274 (2)	C7—H7	0.9300
O1W—H1W	0.8402	C10—C11	1.404 (3)
O1W—H2W	0.8401	C10—H10	0.9300
O2—C8	1.233 (2)	C11—C12	1.362 (4)
O2W—H3W	0.8400	C11—H11	0.9300
O2W—H4W	0.8400	C12—C13	1.392 (4)
O3—C9	1.265 (2)	C12—H12	0.9300
O3—Ni1^ii^	2.0241 (14)	C13—C21	1.404 (3)
O4—C9	1.244 (2)	C13—C14	1.441 (4)
N1—C7	1.323 (3)	C14—C15	1.345 (5)
N1—C5	1.396 (3)	C14—H14	0.9300
N2—C7	1.335 (3)	C15—C16	1.418 (4)
N2—C4	1.376 (3)	C15—H15	0.9300
N2—H2	0.8600	C16—C17	1.400 (5)
N3—C10	1.319 (3)	C16—C20	1.413 (3)
N3—C21	1.364 (3)	C17—C18	1.346 (5)
N5—C19	1.334 (3)	C17—H17	0.9300
N5—C20	1.350 (3)	C18—C19	1.393 (4)
C1—C6	1.381 (3)	C18—H18	0.9300
C1—C2	1.417 (3)	C19—H19	0.9300
C1—C8	1.510 (3)	C20—C21	1.431 (3)
C2—C3	1.384 (3)		
			
O3^i^—Ni1—N5	174.88 (7)	C1—C6—C5	118.45 (17)
O3^i^—Ni1—N3	94.83 (7)	C1—C6—H6	120.8
N5—Ni1—N3	80.31 (8)	C5—C6—H6	120.8
O3^i^—Ni1—N1	91.71 (6)	N1—C7—N2	113.37 (18)
N5—Ni1—N1	90.62 (8)	N1—C7—H7	123.3
N3—Ni1—N1	98.62 (7)	N2—C7—H7	123.3
O3^i^—Ni1—O1W	92.03 (6)	O2—C8—O1	124.26 (18)
N5—Ni1—O1W	92.56 (7)	O2—C8—C1	118.08 (18)
N3—Ni1—O1W	169.42 (6)	O1—C8—C1	117.48 (17)
N1—Ni1—O1W	89.19 (6)	O4—C9—O3	125.86 (19)
O3^i^—Ni1—O2W	85.71 (6)	O4—C9—C2	118.28 (17)
N5—Ni1—O2W	92.07 (7)	O3—C9—C2	115.85 (16)
N3—Ni1—O2W	83.24 (7)	N3—C10—C11	122.1 (2)
N1—Ni1—O2W	176.95 (6)	N3—C10—H10	119.0
O1W—Ni1—O2W	89.27 (6)	C11—C10—H10	119.0
Ni1—O1W—H1W	109.5	C12—C11—C10	119.2 (3)
Ni1—O1W—H2W	98.3	C12—C11—H11	120.4
H1W—O1W—H2W	109.2	C10—C11—H11	120.4
Ni1—O2W—H3W	107.8	C11—C12—C13	120.7 (2)
Ni1—O2W—H4W	102.0	C11—C12—H12	119.6
H3W—O2W—H4W	110.4	C13—C12—H12	119.6
C9—O3—Ni1^ii^	126.97 (12)	C12—C13—C21	116.6 (2)
C7—N1—C5	104.75 (16)	C12—C13—C14	124.8 (3)
C7—N1—Ni1	122.19 (14)	C21—C13—C14	118.6 (3)
C5—N1—Ni1	133.06 (13)	C15—C14—C13	121.1 (3)
C7—N2—C4	107.40 (17)	C15—C14—H14	119.5
C7—N2—H2	126.3	C13—C14—H14	119.5
C4—N2—H2	126.3	C14—C15—C16	121.8 (3)
C10—N3—C21	118.60 (19)	C14—C15—H15	119.1
C10—N3—Ni1	129.35 (15)	C16—C15—H15	119.1
C21—N3—Ni1	112.04 (15)	C17—C16—C15	125.1 (3)
C19—N5—C20	118.7 (2)	C17—C16—C20	116.3 (3)
C19—N5—Ni1	128.25 (18)	C15—C16—C20	118.6 (3)
C20—N5—Ni1	112.85 (15)	C18—C17—C16	121.1 (3)
C6—C1—C2	121.47 (17)	C18—C17—H17	119.5
C6—C1—C8	116.26 (16)	C16—C17—H17	119.5
C2—C1—C8	122.22 (17)	C17—C18—C19	119.2 (3)
C3—C2—C1	120.47 (18)	C17—C18—H18	120.4
C3—C2—C9	118.32 (17)	C19—C18—H18	120.4
C1—C2—C9	121.09 (16)	N5—C19—C18	122.2 (3)
C4—C3—C2	117.13 (18)	N5—C19—H19	118.9
C4—C3—H3	121.4	C18—C19—H19	118.9
C2—C3—H3	121.4	N5—C20—C16	122.5 (2)
N2—C4—C3	131.06 (18)	N5—C20—C21	117.47 (19)
N2—C4—C5	105.66 (17)	C16—C20—C21	120.0 (2)
C3—C4—C5	123.27 (18)	N3—C21—C13	122.9 (2)
N1—C5—C6	131.99 (17)	N3—C21—C20	117.3 (2)
N1—C5—C4	108.82 (16)	C13—C21—C20	119.8 (2)
C6—C5—C4	119.16 (18)		

Symmetry codes: (i) *x*, −*y*+1/2, *z*+1/2; (ii) *x*, −*y*+1/2, *z*−1/2.

### Hydrogen-bond geometry (Å, °)

**Table:**

*D*—H···*A*	*D*—H	H···*A*	*D*···*A*	*D*—H···*A*
O1W—H1W···O1^iii^	0.84	1.90	2.710 (2)	163
O1W—H2W···O4^i^	0.84	1.76	2.584 (2)	165
O2W—H3W···O1^iii^	0.84	1.87	2.703 (2)	169
O2W—H4W···O1^i^	0.84	2.11	2.932 (2)	165
N2—H2···O2^iv^	0.86	2.00	2.739 (2)	144
N2—H2···O1^iv^	0.86	2.54	3.355 (2)	159
C10—H10···O2^i^	0.93	2.56	3.346 (8)	143

Symmetry codes: (iii) −*x*+1, *y*+1/2, −*z*+3/2; (i) *x*, −*y*+1/2, *z*+1/2; (iv) *x*+1, −*y*+1/2, *z*+1/2.
